# Health Perceptions and Practices of a Telewellness Fitness Program: Exploratory Case Study

**DOI:** 10.2196/50710

**Published:** 2024-11-21

**Authors:** Veronica Ahumada-Newhart, Taffeta Wood, Noriko Satake, James P Marcin

**Affiliations:** 1 School of Medicine, University of California Davis, Center for Health and Technology, Sacramento, CA, 95817, United States, 19167342351

**Keywords:** telehealth, telewellness, online fitness, health equity, community health, group exercise, mobile phone, mobile device

## Abstract

**Background:**

During the COVID-19 pandemic, many people lacked access to group fitness opportunities due to elevated risk of infection, lockdown, and closure of exercise facilities. Additionally, many people experienced higher than average rates of mental health burden (eg, anxiety and stress). To help address these needs, an existing in-person community exercise class, taught by a faculty member from an academic medical center, transitioned to an online synchronous (OS) physical fitness class via the Zoom (Zoom Video Communications) videoconferencing platform. As such, the instructor advertised the OS fitness classes through an existing email list of community members and university faculty, staff, students, or alumni email listservs. This telewellness intervention sought to create a sense of community, build social support, and promote physical and mental wellness during the COVID-19 pandemic.

**Objective:**

Our aim was to determine the perceived mental and physical health benefits of attending an OS fitness class for community members, including health care workers. We also assessed the use and functionality of related technologies necessary for delivering and attending the fitness classes.

**Methods:**

An online survey questionnaire was created and tested to collect quantitative and qualitative data for an exploratory study. Data were collected to evaluate the fitness class, motivation, perceived health benefits, and related technologies. A convenience sample of people who had participated in the OS fitness classes was recruited for this study via an emailed recruitment flyer.

**Results:**

A total of 51 participants accessed and completed the survey questionnaire. Survey participants consisted of 28 of 51 (55%) with a university affiliation, 17 of 51 (33%) with no university affiliation, and 6 of 51 (12%) who declined to state. The largest group of participants reporting full-time employment (18/51, 35%) also reported university affiliation with the academic medical center. In this group, 13 of 51 (25%) participants reported full-time employment, university affiliation, and doctoral degrees. High overall exercise class satisfaction was observed in the survey responses (mean 4.0, SD 1). Data analyses revealed significant perceived value of both mental and physical health benefits as motivating factors for participating in the OS fitness class. Challenges were identified as not being able to receive individual feedback from the instructor and the inability of some participants to see if they were in sync with the rest of the class.

**Conclusions:**

Results provide preliminary support for the use of online videoconferencing fitness platforms to promote wellness and facilitate group exercise in the community during times of high infection risk. Future studies should continue to explore perceived benefits, mental and physical wellness, best practices, and the design of related technologies.

## Introduction

### Overview

Regular exercise provides physical and mental health benefits [1,2] including lower cardiovascular disease and lower rates of depression and anxiety [3]. However, many people in the United States do not get sufficient exercise [4]. For many, there are barriers to exercise due to health conditions, financial challenges, or location and limited access to exercise facilities [5]. Virtual fitness classes and other technology-based exercise options are increasingly popular options due to their convenience and accessibility [3]. Online exercise options allow certain groups, such as people living with disabilities and those at elevated risk of infection, to benefit from exercise that they could not otherwise access [6-8]. During the COVID-19 pandemic, people were restricted from social in-person gatherings affecting both physical and mental wellness. Of note, health care workers experienced higher than average rates of burnout due to physical (eg, workload and infection risk) and mental (eg, anxiety and stress) burdens [9-12]. As a result, during the pandemic, many people turned to online exercise options, both asynchronous and synchronous [3].

### Asynchronous and Synchronous Classes

Asynchronous learning is any type of education, instruction, or learning mechanism that does not take place in the same place or at the same time. Asynchronous classes are not confined to a specific schedule. A drawback of asynchronous classes is the lack of live group interaction and experience. It does not provide the opportunity for learners to practice their skills alongside their peers and instructor in real time. Synchronous learning refers to any form of instruction, learning, or education that takes place at the same time but not in the same place. In the context of an online program, synchronous classes are remote and abide by a set day or time schedule and learners can log in from anywhere. In a synchronous program, classes are “live” and happen in real time. Learners are expected to log in and participate at a given time. Questions, discussions, and instruction take place “right then and there” [13]. Synchronous online exercise programs are a promising intervention for adolescents and adults experiencing different health challenges. Researchers have found that synchronous online physical education classes had a positive effect on the improvement of muscle mass, ankle strength (dorsiflexion), hip strength (abduction, flexion, extension, and external rotation), knee strength (extension and flexion), and balance (Y-balance test) in adolescents [14]. Additionally, earlier studies found that synchronous online delivery of exercise classes may provide an accessible alternative for those living in rural or remote locations, as well as for those who may be immunocompromised and cannot attend in-person classes [15]. In adults with >25 BMI, participants who completed synchronous online exercise programs lost significantly more weight (1.8 kg) than those in the waitlist control group (0.25 kg) [16].

Synchronous learning and programs hold great potential for telewellness efforts in the field of telehealth. In our study, we operationalize telewellness as a technology-enabled practice that addresses the social determinants of health with a focus on technology-mediated physical health, mental health, and well-being [17]. Prior research on telewellness has included exercise, nutrition, and mindfulness as promising areas for telewellness interventions [18]. The name of our online synchronous (OS) fitness program, “Zoomba,” was created by the Zumba-certified instructor for phonetic familiarity with the words Zoom and Zumba as dance moves were incorporated into the fitness class. The classes were advertised through the instructor’s personal and work (ie, university) email groups. Classes took place on Mondays and Wednesdays (6 PM) and Saturdays (9 PM), local time, and each class was 1 hour in duration. The exercise level was basic Zumba.

### Telewellness Efforts May Be Extended Through Digital Means such as Videoconferencing Platforms

Videoconferencing platforms hold promise for delivering effective OS fitness classes [19]. Previous research has found the experience of a university-based OS fitness class to be acceptable and enjoyable [20]. Furthermore, digital platform users were more likely to meet physical activity guidelines than nonusers during the COVID-19 pandemic, suggesting that digital platforms could play a role in supporting physical activity [21]. Previous studies have also shown mental health benefits from OS exercise classes. A retrospective study of a wellness program for people with disabilities that provided instruction regarding mindfulness and nutrition, in addition to exercise classes, was found to be feasible and potentially effective in improving several areas of wellness, including mental wellness [22]. A pilot study on the use of a Tai Chi–based exercise combining mind-body exercise and strength training for older adults using wheelchairs found potential benefits for general physical and mental health and proposed delivering these exercises through Zoom [23]. Another study, involving pregnant women, noted that prenatal group fitness classes may improve mental health and found that social connection with other pregnant women in OS fitness classes positively affected mental health [24]. Prior studies have examined the physical health benefits of OS exercise classes, which have shown benefits for cardiovascular health [25]. A clinical study of cardiovascular parameters that compared online exercise training delivered through Zoom to in-person found that both delivery methods provided similar benefits, increased strength and muscle mass, and decreased blood pressure and arterial stiffness [25]. Other studies have examined the feasibility and effectiveness of delivering OS fitness classes to individuals with certain health conditions who benefit from exercise, including children on the autism spectrum [26], adults with Down syndrome [27], adults after bariatric surgery [28], and postpartum women with abdominal muscle separation [29]. Each of these studies used the Zoom platform to provide online exercises specifically designed for the target group. These exercises effectively increased physical activity in children with autism and adults with Down syndrome [27]. Online exercise classes also compared favorably to in-person classes for adults after bariatric surgery [28]. While there are reports regarding the benefits of online exercise for individuals with health conditions, there are very few studies assessing the benefits for general population individuals.

### Fitness Technologies

A wide variety of technology-assisted exercise options exist, including videoconferencing (synchronous) [30], wearable fitness trackers [31], social media, fitness applications [32,33], and online asynchronous recorded exercise content [21]. OS fitness classes have been offered by gyms and fitness studios through various digital platforms [3]. Zoom is commonly used for OS fitness classes [25,26,29], but other technologies may also be used as part of OS fitness classes. For example, Fitbit (Google) wristbands have been used to monitor heart rate during exercise [27]. In addition, WhatsApp (Meta Platforms) groups have been used to organize classes, provide information, and answer questions [26,28]. Previous studies of OS fitness classes have used the telerehab website or a specialized cardiac rehabilitation computer application to facilitate OS fitness classes or provide guidance [34,35].

Our study deployed a similar approach to assess the health benefits of an OS fitness class but instead of evaluating the fitness classes for patients, our study focused on the general population. As the goal of Zoomba was to promote wellness in the community, the classes were advertised to the instructor’s personal and work (ie, university) associated groups. The Zoom platform was selected as it provided access to a fitness class without restrictions related to fitness center fees, childcare, family commitments, transportation, or risk of infection. The OS fitness class model was also selected to create opportunities for reduced feelings of social isolation during the pandemic. Our study contributes findings from an innovative telehealth approach to facilitate health benefits and increase social support.

### Objectives

Our aim was to determine the perceived mental and physical health benefits of attending an OS fitness class. We also assessed the use and functionality of related technologies necessary for delivering and attending the fitness classes.

## Methods

### Study Population and Eligibility

We used nonprobability sampling for this exploratory study as study participants were a convenience sample of community members, including health care workers, who had participated in the Zoomba class and whose email addresses were available to the instructor. To be eligible, the study required participants to be 18 years of age or older; provide confirmation that they had participated in at least 1 Zoomba class; and have access to a computer, tablet, or smartphone with internet or cellular connection.

### Recruitment

A recruitment flyer was emailed to 75 adult participants (18 years of age or older) who had participated in the Zoomba class and had shared their email addresses with the instructor prior to November 1, 2022. Participants were able to access the online study information sheet via a link and QR code on the recruitment flyer. The study information sheet provided university institutional review board–approved information about the study. Agreement to understand the study information sheet and consent to participate were required before participants were able to access the survey questionnaire. All recruitments were completed by December 31, 2022. Participants did not receive compensation for participating. Of the 75 people who received the recruitment flyer, 51 (68%) participants accessed and completed the online questionnaire.

### Procedures

We conducted a retrospective, cross-sectional study, mixed methods design in which an online survey questionnaire was created to collect quantitative and qualitative data [36]. Surveys typically involve quantitative closed-ended items (eg, Likert-scale and multiple-choice) but may include qualitative open-ended questions as well (ie, open text boxes) [37] to effectively combine quantitative and qualitative data to form a mixed methods study [38]. Additionally, to allow participants the freedom to express their views and obtain richer data, our study’s qualitative open-ended questions allowed for an unlimited length of text entries.

### Content Development

We used social cognitive theory to help design the research inquiry and guide the development of the survey. Social cognitive theory posits that cognitive-perceptual factors (perceived benefits, barriers, and self-efficacy) influence engagement in health-promoting behaviors [39,40]. After meeting to discuss the aspects of the class and potential areas of inquiry, 3 of the researchers designed, created, and pretested the survey (VA, TW, and NS).

### Survey Questionnaire

The survey included questions about the physical environment, interactive technologies, and participant demographics to align with previous research on interactive technologies for improved health behaviors. Researchers created a web-based 31-item survey to assess 3 key areas linked to health-promoting behaviors—motivation, perceived health benefits, and self-efficacy. As the fitness classes took place via an online platform, survey questions also included satisfaction with meeting fitness and health expectations, using technologies, and moving into home or work environments during exercise. Prior to deployment, 2 of the researchers tested the survey for functionality and logical progression before distribution of the recruitment flyers. The online survey questionnaire was accessed via the Qualtrics (Qualtrics International Inc) platform. Participants were asked to complete a survey rating their motivation, perceived health benefits, self-efficacy, facilitators or barriers, and satisfaction with the OS fitness class, technologies, and environment. Participants voluntarily completed the anonymous and web-based survey. The survey took approximately 10‐15 minutes to complete.

### Demographic Questionnaire

The demographic questionnaire collected personal information, including age, gender, educational level, university affiliation, and employment status. Additionally, the demographic questionnaire piloted qualitative demographic questions that allowed race or ethnicity to be reported with open-ended text boxes. By allowing participants to self-report race or ethnicity in a language that was comfortable for participants, we hoped to ease the cognitive load from participating in a research study and facilitate data collection that allowed the research team to participate in a 2-way transfer of knowledge. Open-ended qualitative demographic questions may allow for more in-depth learning of identity concepts from participants compared to closed-ended questions with predetermined boxes and labels. For consistency within the academic literature, researchers coded and grouped demographic responses to align with race or ethnicity categories identified by state agencies (see description in Analysis section).

### Ethical Considerations

Ethics approval was obtained from the University institutional review board board (1755525‐1). Informed consent was obtained online from all participants prior to participating. The confidentiality of the participants was maintained at all times. Participants did not receive any compensation for their participation.

## Results

### Survey Results

A total of 51 participants accessed and completed the survey questionnaire. Survey participants consisted of 28 of 51 (55%) with a university affiliation, 17 of 51 (33%) with no university affiliation, and 6 of 51 (12%) who declined to state. The largest group of participants reporting full-time employment (18/51, 35%) also reported university affiliation with the academic medical center. In this group, 13 of 51 (25%) participants reported full-time employment, university affiliation, and doctoral degrees. High overall exercise class satisfaction was observed in the survey responses (mean 4.0, SD 1).

Our study found that the motivation for participating in the OS fitness classes was the highest for safety and remaining accountable for meeting fitness goals. Opportunities to learn new things and socialize were also reported as strong motivators. Perceived health benefits were reported by the majority of study participants with an even divide between physical and mental health benefits. Perceived facilitators and barriers to attending an OS fitness class were also reported. Our study found that the greatest facilitators were stress relief, highly engaging class material, and the ability to see the instructor. Identified barriers were not being able to receive individual feedback and not being able to see if personal movement was in sync with others in the group. As technology played a central role in the OS fitness class, we also collected data on satisfaction with existing technologies used for the class and recommendations for improved technologies and practices.

### Motivation and Perceived Benefits

A total of 46 of 51 (90%) participants reported “staying safe” and 40 of 51 (78%) reported “staying accountable” to fitness goals as the strongest motivating factor for participation in the classes. Learning new things and social aspects of class attendance were also reported as motivators, but with fewer participants—32 of 51 (63%) and 29 of 51 (56%, respectively). In total, 10 of 51 (20%) participants selected “other” for motivation and reported the following motivators*—*fun (n=3), like the instructor (n=2), enjoying Latin music (n=1), curiosity (n=1), support workplace initiative (n=1), easy with children at home (n=2). Participants were allowed to select more than 1 motivating factor and were also provided the opportunity to write their own answers.

Table 1 details participants’ perceived health benefits from attending the OS fitness class. Participants were allowed to select more than 1 response for perceived health benefits. Perceived benefits to both cardiovascular and mental health were reported equally by 46 of 51 (90%) participants for each benefit. Musculoskeletal health was also ranked highly as a perceived health benefit by 44 of 51 (86%) participants. All 44 participants who reported musculoskeletal health as a health benefit also reported cardiovascular and mental health as benefits. Improved sleep and focus were selected by 27 of 51 (53%) and 22 of 51 (43%) participants, respectively. Improved mood, weight loss, learning, and good feelings were also reported as health benefits, with 1 participant stating:

*I feel more flexibility in the way my spine can twist. Feel lighter on my feet. It makes me so happy to move and feel a little bit silly and feel like a kid again*.

Participants were allowed to select more than 1 perceived health benefit and were also provided the opportunity to write their own answers.

A total of 47 of 51 (92%) participants reported their educational attainment. Respondents of various educational attainment levels listed cardiovascular health as a perceived health benefit. In total 18 of 18 (100%) participants with doctoral degrees and 19 of 22 (86%) participants with college or other degrees, compared to 4 of 7 (57%) of participants with master’s degrees, reported cardiovascular health as a perceived health benefit for attending the OS fitness classes. By comparison, mental health had a more consistent distribution as a perceived health benefit among participants from all educational levels. There were 16 of 18 (88%) participants with doctoral degrees, 7 of 7 (100%) participants with master’s degrees, and 18 of 22 (82%) participants with college or other degrees reported mental health as a perceived health benefit of the OS fitness class.

**Table 1.** Participant reported perceived health benefits (N=51).

	Total responses, n (%)	
Cardiovascular health	46 (90)	
Mental health	46 (90)	
Musculoskeletal health	44 (86)	
Improved sleep	27 (53)	
Improved focus	22 (43)	
Other	4 (8)	

### Self-Efficacy and Facilitators or Barriers

Results are reported with the mean and SD for each variable, with possible values ranging from 1 to 5 (Table 2). The lowest variation in responses was reported for relieving stress, engaging class instruction, and being able to see the instructor clearly. However, the highest variability in responses was seen in achieving physical goals, receiving individual feedback, and being able to see if they were moving in sync with the rest of the class. Participant satisfaction, as recorded in the surveys regarding expectations, class instruction, and technology, was high. Expectations being met, the quality of the class instruction, and the quality of the technology used by both instructor and participants were all rated relatively high on a 5-point Likert scale, with most ratings being in the 4 range.

**Table 2.** Participant self-efficacy and facilitators or barriers (N=51).

Participants	Response, mean (SD)
**Expectations: Please rate the following aspects of attending the Zoomba class:**	
Helped me achieve my physical goals	4.2 (0.98)
Helped me feel socially connected	4.3 (0.79)
Helped me relieve stress	4.5 (0.73)
**Class instruction: Please rate the following aspects of the Zoomba class instruction:**	
Class instructions were clear to me	4.5 (0.61)
Class was engaging	4.7 (0.46)
Class was the right level of difficulty for me	4.4 (0.77)
Instructor was able to provide me with individual feedback	3.5 (1.2)

### Technology Use

Sessions were delivered by the instructor using a laptop, video, audio, and music for the sessions were shared via the Zoom platform. Participants reported using laptop computers, smartphones, tablets, and desktop computers to attend the classes. Overall, participants reported high satisfaction with the technical capabilities to hear and see the instructor clearly. Sessions were delivered by the instructor using a laptop, video, audio, and music for the sessions shared via the Zoom platform. Participants reported using laptops, smartphones, tablets, and desktop computers to attend the classes. Participants rated satisfaction with technology on a 5-point Likert scale, with 1 being the lowest satisfaction and 5 being the highest satisfaction. Overall, 51 participants reported high satisfaction with the technical capabilities to hear and see the instructor clearly. “I was able to hear the instructor clearly” and “I was able to see the instructor clearly” received response mean of 4.3 (SD 0.90) and 4.4 (SD 0.48), respectively. “I was able to see if I moved in sync with others” received a lower mean of 3.3 (SD 1.36).

### Technology Recommendations

When asked if there was anything that could be improved in the technology, 9 participants provided several recommendations (Table 3).

**Table 3.** Technology recommendations.

Participant #	Technology used	Camera on during class	Recommendation
**Recommendations for the instructor:**			
1	Laptop	Yes	Better AV (audio or visual equipment)
2	Tablet	No	Better microphone
3	Tablet	No	Using a microphone to instruct next steps or advise. I could hear instructor’s instruction only when music is quiet.
4	Laptop	Yes	Probably more investments for instructor to do in a larger scale.
5	Laptop	Yes	...having a motion-capture virtual avatar would help for people who are camera shy
**Recommendations for remote participants:**			
6	Laptop	Yes	Bigger screen (at home) to see the others better
7	Laptop	Yes	I just have to make sure our Wi-Fi is working properly.
8	Laptop and headphones	Yes	Ask participants who appear on camera to set Zoom to mirror their image. Then we might be all in sync—a synchronized zoom challenge could be fun!
9	Laptop	Yes	More people turning on their cameras! (If they’re comfortable). In other Zumba classes, the instructor will briefly go over trickier steps before the song starts so everyone can keep up
5 (continued)	Laptop	Yes	More audience participation (more cameras on)...

Requests such as, *“*a bigger screen to see the others better*”* and

*ask participants who appear on camera to set Zoom to mirror their image. Then we might be all in sync – a synchronized zoom challenge could be fun!*

Suggested that participants viewed this use of technology to support a need for socialization with the fitness group.

General recommendations for the “Zoomba” class included suggestions for music, moves, and strength exercises. Two participants recommended that the class be better advertised, with 1 participant specifying, “Offering more outside of UC Davis as this helps a lot of people’s mental health.”

### Barriers to Participation

Additionally, the survey included opportunities for participants to report any experienced barriers to remote exercise. A total of 15 people reported the following challenges at home, with some participants reporting more than 1 challenge. Additionally, the survey included opportunities for participants to report any experienced barriers to remote exercise. In total, 15 participants reported barriers to participation in their homes, in which 10 people reported challenges with furniture; 9 people reported challenges with others (eg, children, adults, and pets); and 1 person reported challenges with home Wi-Fi. Some participants reported more than 1 barrier to participation.

### Education and Employment

Participants reported high levels of employment and education, with 28 of 51 (55%) reporting affiliation with the academic medical center (ie, university affiliation) (Table 4). The largest group of participants reporting full-time employment (18/51, 35%) were affiliated with the academic medical center. In this group, 13 of 51 (25%) participants reported full-time employment, university affiliation, and doctoral degrees. The smallest group of participants were part-time employees, affiliated with the academic medical center, 3 of 51 (6%), with varying educational levels. As there were several degree options on our form, we grouped associates (n=4), bachelors (n=21), and “other” (n=1) degrees in the “College/Other” category.

**Table 4.** Participant demographics by employment status and university affiliation (UA).

	UA, n	No UA, n	Decline affiliation, n	College or other (n=22), n		Masters (n=7), n		Doctorate (n=18), n		Decline education (n=4), n	
				No UA	UA	No UA	UA	No UA	UA	No UA	UA
Full-time	18	13	1	8	3	1	2	4	13	1	—^a^—
Part-time	3	0	0	0	1	0	1	0	1	0	—
Student	5	1	0	0	3	0	2	0	0	1	—
Not employed	0	0	4	4	0	0	0	0	0	0	—
Decline to answer	2	3	1	2	1	1	0	0	0	1	1
Total	28	17	6	14	8	2	5	4	14	3	1

a— : not available.

### Race or Ethnicity and Gender

Our study piloted qualitative demographic questions that allowed race or ethnicity to be reported with open-ended text boxes to ease the cognitive load and facilitate data collection that was representative of participant identities. Researchers coded and grouped self-reported demographic responses to align with guidelines from the State Department of Public Health on race or ethnicity. In Table 5, the Asian population group includes participants who self-identified as “Asian,” “Filipino,” “Asian/Pacific Islander,” “South Indian,” “Middle Eastern,” and “Japanese.” The multirace population group includes participants who self-identified as “Caucasian/Asian,” “Multiple Races,” “White/Persian,” and “White + African American descendent.”

**Table 5.** Participant demographics by age group and university affiliation (UA).

	Male (n=6), n		Female (n=40), n		Decline gender (n=5), n		Asian (n=24), n		Black (n=2), n		White (n=11), n		Multi (n=7), n		Decline race or ethnicity (n=7), n	
	No UA	UA	No UA	UA	No UA	UA	No UA	UA	No UA	UA	No UA	UA	No UA	UA	No UA	UA
18‐24	0	0	0	3	1	0	0	2	0	0	0	0	0	1	1	0
25‐34	1	0	0	5	0	0	1	2	0	1	0	0	0	1	0	1
35‐44	1	0	3	4	1	0	2	3	1	0	1	1	0	0	1	0
45‐54	0	2	4	6	0	0	2	2	0	0	1	3	0	1	1	2
55‐64	0	1	5	3	1	0	3	2	0	0	1	2	1	0	1	0
Older than 65	0	1	3	3	0	0	3	2	0	0	1	1	0	0	0	0
Decline age	0	0	0	1	2	0	0	0	0	0	0	0	3	0	0	0
Total	2	4	15	25	5	0	11	13	1	1	4	7	4	3	4	3

## Discussion

### Principal Findings

This study examined the perceived mental and physical health benefits of attending an OS fitness class for community members, including health care workers. Given the use of laptops, tablets, cameras, speakers, and microphones, this study also assessed the use and functionality of these related technologies for delivering and attending OS fitness classes.

In our study, participants reported that the “Zoomba” classes promoted health benefits for physical health (cardiovascular and musculoskeletal), mental health, and cognitive function (improved sleep and improved focus). This suggests that participants have different motivations for participating in class and may benefit across both physical and mental health domains. The relatively low technical and financial investment for such benefits may represent an effective and accessible delivery model of telewellness programs for general populations. Findings from our study are consistent with past interventions that demonstrate high remote engagement through videoconferencing [7,41,42]. Telehealth interventions that deliver synchronous group-based exercise sessions for populations seeking physical and mental health benefits constitute a promising avenue for future research in the post-pandemic era.

Findings from this research can be applied to the online delivery of exercise classes supported by the workplace, community organizations, or other populations where a sense of safety and social support may contribute to participation in group fitness classes. In this study, 13 of 51 (25%) participants reported full-time employment, university affiliation, and doctoral degrees. As the health care workforce may experience longer work hours at times of high risk of infection, transitioning in-person fitness classes to OS fitness classes may provide flexibility and increased safety for those most heavily impacted during times of high risk of infection.

Results from this study reinforce that perceived health benefits, social supports, and a sense of community may be fostered in OS fitness class environments. These supports may be viewed as facilitators for attending OS fitness classes. However, barriers that may limit full engagement in OS fitness classes were also related to the social aspects of the class. Areas with the lowest satisfaction were (1) the ability to receive individual feedback from the instructor and (2) the ability for participants to see if they were moving in sync with others. Findings related to these social disconnects and the importance of social interaction are consistent with other studies of OS fitness classes during the COVID-19 pandemic [43]. Future studies may explore increased social interactions both pre- and postclass to meet these needs and increase support for adherence to fitness goals for health benefits. A deeper understanding of motivation, social supports, and the impact of OS fitness classes may benefit population groups that lack access and resources to attending group fitness classes in high-cost gyms or classes.

### Comparison to Prior Work

While earlier studies have focused on specific populations including pregnant women [26], those with developmental disabilities [27], or patients post bariatric [28], our study evaluated an OS fitness class during the COVID-19 pandemic for the general population. Our study contributes a focus on community members, including health care workers, during a time of extremely high infection risk and health care workforce shortages. As rates of health care worker burnout are alarmingly high, interventions that provide social support, health benefits, and social connections are urgently needed. Research has highlighted the need for low-cost institutional and personal interventions [44]. Innovative, low-cost, strategies like the Zoomba classes are essential for the successful implementation of telewellness interventions to meet the needs of general populations.

### Strengths and Limitations

Some limitations of this study include generalizability, small sample size, and postintervention survey. First, our findings may not be generalizable to all communities as our study participants represented a well-educated (majority college-educated), majority female population. Second, our sample size was small at 51 participants and recruitment was limited to the instructor’s email lists. Second, in designing the survey, we did not include questions on participants’ employment status as health professionals, such as nurses or medical doctors. This may have limited our ability to draw insights on patterns or participant ratings of health benefits for specific employee groups in an academic medical center, university system, or the local community. Third, as the survey was completed months after the intervention, the lapse in time between the class starting and the questionnaire being distributed may have influenced participants’ views of the experience. With many fitness centers being closed during the COVID-19 pandemic, the OS fitness classes may have been more generously rated than future OS exercise offerings. Additionally, the survey was reliant on the participant’s self-report versus other quantifiable health measures. However, such self-selection is present in most survey studies.

### Future Directions

Future studies will explore OS fitness class design to promote and sustain health benefits, assess motivation, and social support. Longitudinal studies may reveal more complex relationships between telehealth technologies used for fitness and social support. Additionally, future studies will explore collecting quantifiable health data (eg, weight, blood pressure, resting heart rate, perceived well-being, and belonging) as related to participant motivation and related fitness goals.

### Conclusions

Our findings confirm that OS fitness classes are effective in promoting health behaviors for groups hesitant to attend in-person classes in times of high infection risk. The perceived health benefits were equally divided between cardiovascular and mental health. Participant ratings on met expectations, class instruction, and effectiveness of technologies were generally high (mean scores of 4+ on a 5-point Likert scale). Participants reported lower satisfaction with the ability to receive individual feedback and being able to see and move in sync with others. Results suggest a need for research on the design of digital health technologies to facilitate these challenges. Additionally, our findings suggest that community-focused behavioral health interventions that support individuals with safety concerns may effectively contribute to wellness outcomes. The use of interactive technologies to connect individuals with similar health interests may also facilitate improved health behaviors. Results suggest a continued need for research on the effectiveness of OS fitness classes that provide health benefits.

## Abbreviations

OS: online synchronous
